# Potential Role and Prognostic Value of Erythropoietin Levels in Patients With Severe Aortic Stenosis Undergoing Transcatheter Aortic Valve Replacement

**DOI:** 10.3389/fcvm.2020.605257

**Published:** 2020-11-30

**Authors:** Silvia Mas-Peiro, Philipp C. Seppelt, Roberta De Rosa, Marie-Isabel Murray, Jörg Yogarajah, Alexander Berkowitsch, Stephan Fichtlscherer, Andreas M. Zeiher, Mariuca Vasa-Nicotera

**Affiliations:** ^1^Department of Cardiology, University Hospital Frankfurt am Main, Frankfurt, Germany; ^2^German Center for Cardiovascular Research (DZHK), Berlin, Germany; ^3^Cardiopulmonary Institute (CPI), Frankfurt, Germany

**Keywords:** prognostic value, biomarker, anemia, transcatheter aortic valve replacement (TAVR), aortic valve stenosis (AS), erythropoietin (EPO)

## Abstract

**Background:** Both EPO levels and anemia have shown prognostic value in several cardiac disorders. An observational study with a prospective follow-up was performed to investigate their independent prognostic roles in severe aortic stenosis.

**Methods:** An up to 36-month follow-up of consecutive patients with severe aortic stenosis undergoing TAVR in a high-volume center was performed. Patients with eGRF <30 mL/min/1.73 m^2^ were excluded. EPO levels and/or anemia status and its association with mid-term mortality were assessed.

**Results:** Out of 407, 360 met eligibility criteria. Median age was 83 years, with 71.4% having a NYHA class III/IV. Anemia was present in 51.9%, and iron deficiency in 52.8%. Median (IQR) EPO levels were 14.4 (9.30–24.30) mIU/mL. Median follow-up was 566 days. Anemia was associated with overall mortality (HR 2.40, 95% CI 1.51–3.80, *p* < 0.001). Higher logEPO levels were associated with mid-term mortality (HR 4.05, 95% CI 2.29–7.16, *p* < 0.001), even after adjusting for clinically and/or statistically relevant factors (multivariate HR 2.25, 95 CI 1.09–4.66, *p* = 0.029). Kaplan-Meier analyses showed early diverging curves for anemia vs. non-anemia, whereas curves for patients in various EPO level quartiles started to diverge at about 100 days, with differences consistently increasing during the subsequent entire follow-up period.

**Conclusions:** Differently from anemia, which was a strong predictor for both early and late mortality in severe aortic stenosis after TAVR, independent prognostic value of EPO only emerged after post-TAVR recovery. EPO prognostic value was independent from anemia and mild-to-moderate renal dysfunction. High EPO levels could be useful to identify patients with severe aortic stenosis showing a compromised mid-term survival in spite of TAVR use and independently from early TAVR results.

## Introduction

Being synthesized in kidneys in response to hypoxic stimuli, erythropoietin (EPO) has a hematopoietic effect on the bone marrow, promoting an increased development of the erythroid lineage and leading to an increased production of erythrocytes. An impaired EPO production that causes anemia, can be found in patients with chronic kidney disease (CKD). Recombinant human EPO (rhEPO) has been successfully used in CKD patients with EPO deficiency (1).

Both anemia and renal dysfunction are commonly present in heart failure (2), and the use of exogenous EPO has been investigated in such patients (2). The findings suggest that EPO administration does not improve clinical outcome (2) and, thus, routine EPO use is not recommended by current guidelines (3, 4). The EPICURE randomized clinical trial, an intervention study of exogenous EPO use, has shown negative results also in patients with severe aortic stenosis (5). In fact, studies of EPO levels in patients with heart failure showed that excessive EPO synthesis predicts mortality in chronic and acute heart failure (6–8), possibly reflecting a status of advanced cardiac disease.

Aortic stenosis remains the most common heart valve disease with a poor prognosis if not treated. Trans-catheter aortic valve replacement (TAVR) has dramatically improved prognosis in selected patients with severe aortic stenosis, particularly in those not eligible for surgical replacement (9). Certain prognostic biomarkers (including those reflecting anemia, renal dysfunction, and malnutrition) (10–13) have been shown to be associated to lower survival rates. Nevertheless, available biomarkers are still limited and there is an unmet need for novel powerful predictors of mortality in such patients. To our knowledge, the impact of endogenous EPO levels on clinical outcomes in severe aortic stenosis has not yet been investigated.

Our study aimed at assessing the prognostic value of baseline endogenous EPO for short- and mid-term survival in patients with severe aortic valve stenosis undergoing TAVR, and whether this is related to or independent from the prognostic impact of anemia and renal function, in patients with preserved renal function.

## Methods

### Study Population and Procedures

All consecutive patients with severe aortic stenosis undergoing TAVR between July 2016 and October 2019 in a high-volume tertiary university hospital were included, after assessment by heart team. Procedures were performed under local anesthesia unless there was a specific reason to use general anesthesia. In TAVR procedures, both balloon-expandable and self-expandable devices were implanted. No patient received exogenous EPO. Patients undergoing a valve-in-valve procedure were excluded from the analysis, in order to focus solely on native degenerative aortic stenosis. Patients with any missing value for baseline anemia, iron deficiency, and erythropoietin assessments were not included. Finally, as suggested for studies of EPO as a potential biomarker, patients with eGRF < 30 mL/min/1.73 m^2^ were also excluded, since erythropoiesis is severely impaired in such specific population.

An up to 36-month prospective follow-up was completed through outpatient visits or telephone interviews with patients or family members.

### Baseline Clinical, Echocardiographic, and General Clinical Chemistry Data

Baseline demographic parameters, previous comorbidities, and baseline echocardiographic parameters were collected on admission. Renal function was measured by means of the estimated glomerular filtration rate (eGFR) using the abbreviated Modification of Diet in Renal Disease equation (14): GFR (ml/min/1.73 m^2^) = 175 × S-Creatinine ^−1, 154^ × Age ^−0, 203^ (×0.742 if female). Chronic kidney disease (CKD) stages were used to classify eGFR: CKD 1 (>90), 2 (60–90), 3 (30–60), 4 (15–30), and 5 (0–15) mL/min/1.73 m^2^. Cardiac biomarkers, including NTproBNP and high-sensitive troponin, and high-sensitive C-reactive protein (hs-CRP), an inflammatory marker, were also measured in all patients. The potential association of EPO levels with other traditional risk factors, including diabetes mellitus, smoker status, and coronary artery disease, as well as with statin use, was specifically explored.

### Baseline Hematology Parameters and Definition of Anemia and Iron Deficiency

Baseline hemoglobin, platelet and white blood cell counts were measured in all patients at the time of admission. WHO guidelines were used to define anemia (Hb < 12 g/dl for women, < 13 g/dl for men) (15). Iron, ferritin, transferrin, and transferrin saturation were assessed. Iron deficiency was defined according to Kidney Disease Outcomes Quality Initiative guidelines either as ferritin levels of < 100 ng/ml or based on the percentage of transferrin saturation (defined as serum iron (μg/dL)/[serum transferrin (mg/dL) × 1.25] < 20% when ferritin is < 800 ng/mL). This dual definition takes into account both functional and absolute aspects of iron deficiency (16). Baseline EPO levels were measured in all patients using a CLIA (Siemens) assay, before TAVR procedure.

In addition to EPO levels, as suggested for studies in anemic patients (17, 18), the observed/predicted ratio was also calculated, with predicted levels being based on hemoglobin values, as per a previously validated formula: 4.640-(0.274 × hemoglobin)=log EPO (7, 18). Limits previously found in a heart failure cohort were used to determine the proportion of patients with a lower- or higher-than-expected EPO production (18).

### Study Endpoints

The main study endpoint was mid-term (up to 36 months) all-cause mortality. Secondary endpoints included 30-day mortality, 1-year mortality, and complications according to Valve Academic Research Consortium 2 (VARC-2) criteria. Causes of in-hospital death were based on the assessment reported by attending physicians; causes of mid-term death were mostly provided by GPs (through interviews with a study physician) and in some cases were based on details obtained from family interviews or could not be ascertained.

### Statistical Analysis

Continuous variables are presented as median (IQR). Frequencies were used to report categorical variables. Patients were split into quartiles of EPO values. Comparisons of baseline parameters between groups (EPO quartiles) were done with Kruskal-Wallis. In case of revealed significance by multi-group test, a Bonferroni correction was also used. Kaplan-Meier survival curves were plotted for EPO median or quartiles, as well as for patients with vs. without baseline anemia as defined by WHO (15). A multivariate Cox regression analysis was performed based on both the variables achieving significance (*p* < 0.05) in univariate regression analyses and those additional variables considered to be clinically relevant for mid-term mortality. Pre-specified subgroup analyses of logEPO prognostic value were performed for patients with and without anemia, and for patients without (CKD 1+2) and with moderate renal dysfunction (CKD 3 stage). Statistical significance was set at *p* < 0.05. All analyses were performed with the SPSS statistical software package version 24.0.

### Ethics

Informed consent was obtained from all patients. The protocol of the present study conforms to the ethical guidelines of the 1975 Declaration of Helsinki and was approved by the ethics committee of the Goethe University Frankfurt am Main, Germany (296/16).

## Results

### Study Population

Out of 563 patients with severe aortic stenosis undergoing TAVR at our center over the study period, a complete baseline profile including anemia status, iron status, and EPO levels was available in 407 patients. With laboratory tests having been routinely ordered in all patients, missing data were due to many different reasons and randomly distributed, with no pattern being observed. Eight patients undergoing a valve-in-valve procedure were excluded from the analysis; and 39 patients with eGFR < 30 mL/min/1.73 m^2^ were also excluded. Our final population with complete data consisted of a total of 360 patients with severe aortic stenosis. In TAVR procedures, both balloon- and self-expandable valves were implanted: 22.2% Sapien 3 (Edwards Lifesciences), 28.6% Symetis Acurate Neo (Boston Scientific), 36.1% Portico (Abbott Vascular), and 13.0% Evolut R (Medtronic). Nearly all procedures were performed under local anesthesia.

### Baseline Characteristics

Overall median (IQR) age was 83 (79–86) and 40.8% were female, with 71.4% having a NYHA functional class III/IV; median (IQR) STS score was 3.17 (2.31–4.90). Overall median (IQR) left ventricular ejection fraction (LVEF) was 60 (50–60) and median (IQR) transvalvular gradient was 43 (33–53) mmHg. Most patients (251 out of 360) were on statins.

Baseline anemia (defined according to WHO criteria) was present in 51.9% of the population; absolute and relative iron deficiency was found in 52.8%. Baseline median (IQR) serum iron levels were 73 (52–96) μg/dl, ferritin levels were 152 (79–279) ng/ml, median transferrin saturation index was 21.54 % (15.14–28.86), and median endogenous EPO levels were 14.4 (9.30–24.30) mIU/mL.

Table 1 shows baseline characteristics and echocardiographic parameters across EPO quartiles; Table 2 shows laboratory values and STS score across EPO quartiles. NYHA class IV status, STS score, previous PCI, and history of COPD were higher in upper EPO quartiles. As for laboratory parameters, NTproBNP values increased across quartiles; and hemoglobin levels, eGFR, and iron levels decreased across EPO quartiles.

**Table 1:** Baseline clinical and echocardiographic parameters across EPO quartiles.

	**Total**	**Q1**	**Q2**	**Q3**	**Q4**	***P*-value**
**EPO range (mIU/ml)**		**≤9.3**	**9.4–14.4**	**14.5–24.2**	**≥24.3**	
***N***	**360**	**91**	**91**	**87**	**91**	
	**N (%)**	**N (%)**	**N (%)**	**N (%)**	**N (%)**	
Age (years)	**83** (79–86)	**82** (79–84)	**83** (78–86)	**83** (79–87)	**83** (79–86)	0.1400
Male (%)	**213** (59.17%)	**59** (64.84%)	**48** (52.75%)	**54** (62.07%)	**52** (57.14%)	0.3589
BMI (kg/m^2^)	**26.50** (24.1–29.6)	**26.0** (23.6–29.3)	**26.4** (24.2–30.1)	**26.8** (24.0–29.8)	**26.5** (24.2–30.1)	0.8150
Carotid occlusive disease (%)	**72** (20.00%)	**20** (21.98%)	**13** (14.29%)	**17** (19.54%)	**22** (24.18%)	0.3790
PAOD (%)	**47** (13.06%)	**16** (17.58%)	**9** (9.89%)	**9** (10.34%)	**13** (14.29%)	0.3720
Previous cardiac surgery (%)	**35** (9.72%)	**10** (10.99%)	**3** (3.30%)	**9** (10.34%)	**13** (14.29%)	0.0840
COPD (%)	**61** (16.94%)	**11** (12.09%)	**15** (16.48%)	**17** (19.54%)	**18** (19.78%)	0.4800
DM (%)	**119** (33.06%)	**29** (31.87%)	**23** (25.27%)	**29** (33.33%)	**38** (41.76%)	0.1290
Insulin dependent DM (%)	**36** (10.00%)	**9** (9.89%)	**4** (4.40%)	**9** (10.34%)	**14** (15.38%)	0.1060
Hypertension (%)	**307** (85.28%)	**74** (81.32%)	**75** (82.42%)	**79** (90.80%)	**79** (86.81%)	0.2500
**NYHA status (%)**						
I	**3** (0.83%)	**1** (1.10%)	**2** (2.20%)	**0** (0.00%)	**0 (**0.00%)	**0.0029**
II	**100** (27.78%)	**33** (36.26%)	**27** (29.67%)	**23** (26.44%)	**17** (18.68%)	
III	**232** (64.44%)	**56** (61.54%)	**60** (65.93%)	**55** (63.22%)	**61** (67.03%)	
IV	**25** (6.94%)	**1** (1.10%)	**2** (2.20%)	**9** (10.34%)	**13** (14.29%)	
Previous MI (%)	**62** (17.22%)	**15** (16.48%)	**10** (10.99%)	**15** (17.24%)	**22** (24.18%)	0.1330
Previous stroke (%)	**48** (13.33%)	**18** (19.78%)	**10** (10.99%)	**8** (9.20%)	**12** (13.19%)	0.1720
Previous TIA (%)	**12** (3.33%)	**4** (4.40%)	**3** (3.30%)	**3** (3.45%)	**2** (2.20%)	0.8810
Previous PCI (%)	**155** (43.06%)	**33** (36.26%)	**35** (38.46%)	**38** (43.68%)	**49** (53.85%)	0.0770
Coronary artery disease (%)	**247** (68.61%)	**61** (67.03%)	**56** (61.54%)	**60** (68.97%)	**70** (76.92%)	0.1620
Previous atrial fibrillation (%)	**166** (46.11%)	**39** (42.86%)	**35** (38.46%)	**42** (48.28%)	**50** (54.95%)	0.1360
LVEF (%)	**60 (**50–60)	**60 (**50–60)	**60 (**53–60)	**60** (50–60)	**55** (40–60)	0.0730
Pmean (mmHg)	**43** (33–53)	**45** (37–60)	**43** (32–54)	**41** (32–49)	**41** (32–51)	0.2370

*COPD, chronic obstructive pulmonary disease; DM, diabetes mellitus; LVEF, left ventricular ejection fraction; MI, myocardial infarction; NYHA, New York Heart Association; PAOD, peripheral arterial occlusive disease; PCI, percutaneous coronary intervention; Pmean: mean transvalvular gradient; TIA, transient ischemic attack. P-values in bold are statistically significant (Kruskal-Wallis test)*.

**Table 2:** Baseline laboratory parameters across EPO quartiles.

	**Total**	**Q1**	**Q2**	**Q3**	**Q4**	***P*-value**	**Bonferroni correction (subgroup - test)**					
							**Q1-Q2**	**Q2-Q3**	**Q2-Q4**	**Q1-Q3**	**Q1-Q4**	**Q3-Q4**
**N**	**360**	**91**	**91**	**87**	**91**							
**EPO range (mIU/ml)**		**≤9.3**	**9.4–14.4**	**14.5–24.2**	**≥24.3**							
	**Median (IQR)**	**Median (IQR)**	**Median (IQR)**	**Median (IQR)**	**Median (IQR)**							
Creatinine (mg/dl)	**1.09** (0.87–1.33)	**1.07** (0.86–1.29)	**1.02** (0.85–1.22)	**1.11** (0.88–1.41)	**1.15** (0.93–1.42)	**0.028**	1.000	0.308	0.029	1.000	0.327	1.000
eGFR (ml/min/1.73m^2^)	**58.80** (46.00–72.70)	**62.10** (46.00–77.00)	**60.90** (51.50–74.90)	**56.20** (45.00–69.20)	**54.7** (41.00–66.1)	**0.007**	1.000	0.332	0.016	0.592	0.037	1.000
**CKD**												
1	**24** (6.67%)	**6** (6.59%)	**8** (8.79%)	**5** (5.75%)	**5** (5.49%)	0.0850						
2	**144** (40.00%)	**46** (50.55%)	**39** (42.86%)	**32** (36.78%)	**27** (29.67%)							
3	**192** (53.33%)	**39** (42.86%)	**44** (48.35%)	**50** (57.47%)	**59** (64.84%)							
Urea (mg/dl)	**44** (34–55)	**46** (35–56)	**41** (34–50)	**44** (33–59)	**43** (36–56)	0.245						
NTproBNP (pg/ml)	**1,701** (790–3,733)	**1,325** (619–2,115)	**1,464** (778–2,736)	**1,823** (688–3,636)	**3,516** (1,236–9,022)	**<0.001**	1.000	1.000	<0.001	0.462	<0.001	0.007
High–sensitive Troponin (pg/ml)	**23** (14–38)	**21** (13–33)	**21** (13–29)	**23** (15–33)	**35** (19–65)	** <0.001**	1.000	1.000	<0.001	1.000	<0.001	0.003
CK (U/l)	**75** (54–113)	**87** (60–116)	**71** (55–105)	**79** (57–109)	**63** (43–118)	**0.044**	0.790	1.000	1.000	1.000	0.030	0.491
CK–MB (U/l)	**18** (14–24)	**19** (15–26)	**19** (15–24)	**16** (13–22)	**16** (13–25)	**0.004**	1.000	0.069	0.226	0.015	0.058	1.000
Hemoglobin (g/dl)	**12.4** (11.0–13.6)	**13.5** (12.6–14.4)	**12.7** (11.9–13.7)	**11.9** (10.9–13.3)	**10.8** (9.6–12.1)	** <0.001**	0.022	0.078	<0.001	<0.001	<0.001	<0.001
Platelets (/nl)	**214.5** (176–262)	**209** (182–247)	**222** (173–255)	**199** (157–253)	**226** (192–300)	**0.019**	1.000	0.769	0.610	1.000	0.382	0.010
Leukocytes (/nl)	**7.10** (5.90–8.34)	**7.26** (5.92–8.52)	**7.17** (6.40–8.39)	**6.84** (5.42–8.30)	**6.78** (5.91–8.34)	0.367						
C–reactive Protein (mg/dl)	**0.30** (0.13–0.78)	**0.16** (0.07–0.38)	**0.28** (0.11–0.59)	**0.32** (0.15–0.75)	**0.78** (0.26–1.77)	** <0.001**	0.103	1.000	<0.001	0.012	<0.001	0.001
Iron (μg/dl)	**73** (52–96)	**88** (68–109)	**80** (57–99)	**75** (55–96)	**48** (34–72)	** <0.001**	0.185	1.000	<0.001	0.026	<0.001	<0.001
Ferritin (ng/ml)	**152** (79–279)	**185** (101–341)	**175** (96–277)	**149** (81–274)	**94** (47–262)	**0.001**	1.000	1.000	0.030	0.277	<0.001	0.239
Transferrin (mg/dl)	**245** (216–277)	**239** (216–273)	**245** (218–269)	**245** (216–274)	**258** (212–302)	0.423						
Transferrin saturation (%)	**21.54** (15.14–28.86)	**25.18** (21.06–32.11)	**23.80** (16.14–29.95)	**21.48** (15.56–28.54)	**15.27** (10.17–20.87)	** <0.001**	0.314	1.000	<0.001	0.043	<0.001	<0.001
STS score	**3.17** (2.31–4.90)	**2.91** (1.87–4.68)	**2.96** (2.20–3.74)	**3.55** (2.46–5.79)	**3.57** (2.70–5.33)	**0.001**	1.000	0.046	0.006	0.069	0.010	1.000

*CK, creatine kinase; CKD, chronic kidney disease; CK-MB, creatin kinase muscle-brain fraction, eGFR, estimated glomerular filtration rate. P-values in bold are statistically significant (Kruskal-Wallis test)*.

A statistically significant (*p* = 0.002) but weak (*r*^2^ = 0.027) correlation between logEPO and LVEF was found. Increasing median EPO levels were observed for lower LVEF categories (Median (IQR) EPO levels [mIU/mL] 13.60 [9.00–22.50], 15.80 [11.40–25.20], 17.80 [8.0–31.40], and 21.20 [10.95–40.50] for LVEF >55%, 45–54, 30–44, and <30%, respectively; *p* = 0.063).

No association was found between EPO levels and other traditional risk factors in both sexes. Median (IQR) EPO levels (mIU/mL) in patients with vs. without diabetes mellitus were 15.20 (12.50–29.90) vs. 13.85 (9.50–23.80) (*p* = 0.097) in female and 16.60 (8.00–28.30) vs. 13.75 (8.95–20.15) (*p* = 0.220) in male. EPO levels in patients with vs. without coronary artery disease were 15.00 (9.95–29.55) vs. 13.55 (9.58–22.60) (*p* = 0.272) and 15.40 (8.95–24.55) vs. 11.65 (8.00–17.60) (*p* = 0.092), respectively. And EPO levels in smokers/former smokers vs. never smokers were 9.30 (8.30–17.10) vs. 14.40 (10.30–24.50) (*p* = 0.290) and 14.70 (8.60–21.50) vs. 14.10 (9.00–24.50) (*p* = 0.424), respectively. No association was found between statin use and EPO levels (median [IQR] EPO values [mIU/mL] in patients with or without statin use, 14.40 vs. 14.20, *p* = 0.893) in our cohort.

### Mortality, Complications, and Impact of EPO Levels, Anemia and Iron Deficiency

The median (min, max) follow-up was 566 (0–1,086) days. Overall mortality was 3.9% at 30 days, and 16.9% at 12 months, with rates increasing across EPO quartiles (8.79% Q1, 13.19% Q2, 21.84% Q3, 24.18% Q4). High logEPO levels on admission were significantly associated with mid-term mortality (HR 4.05, 95% CI 2.29–7.16, *p* < 0.001). Complications according to VARC-2 criteria in the overall population were as follows: permanent pacemaker implantation (15.8%), major cerebrovascular accident (2.5%), minor cerebrovascular accident (0.8%), pericardial tamponade (0.6%), major vascular complication (5.3%), and major bleeding (4.4%).

**Figure 1:**
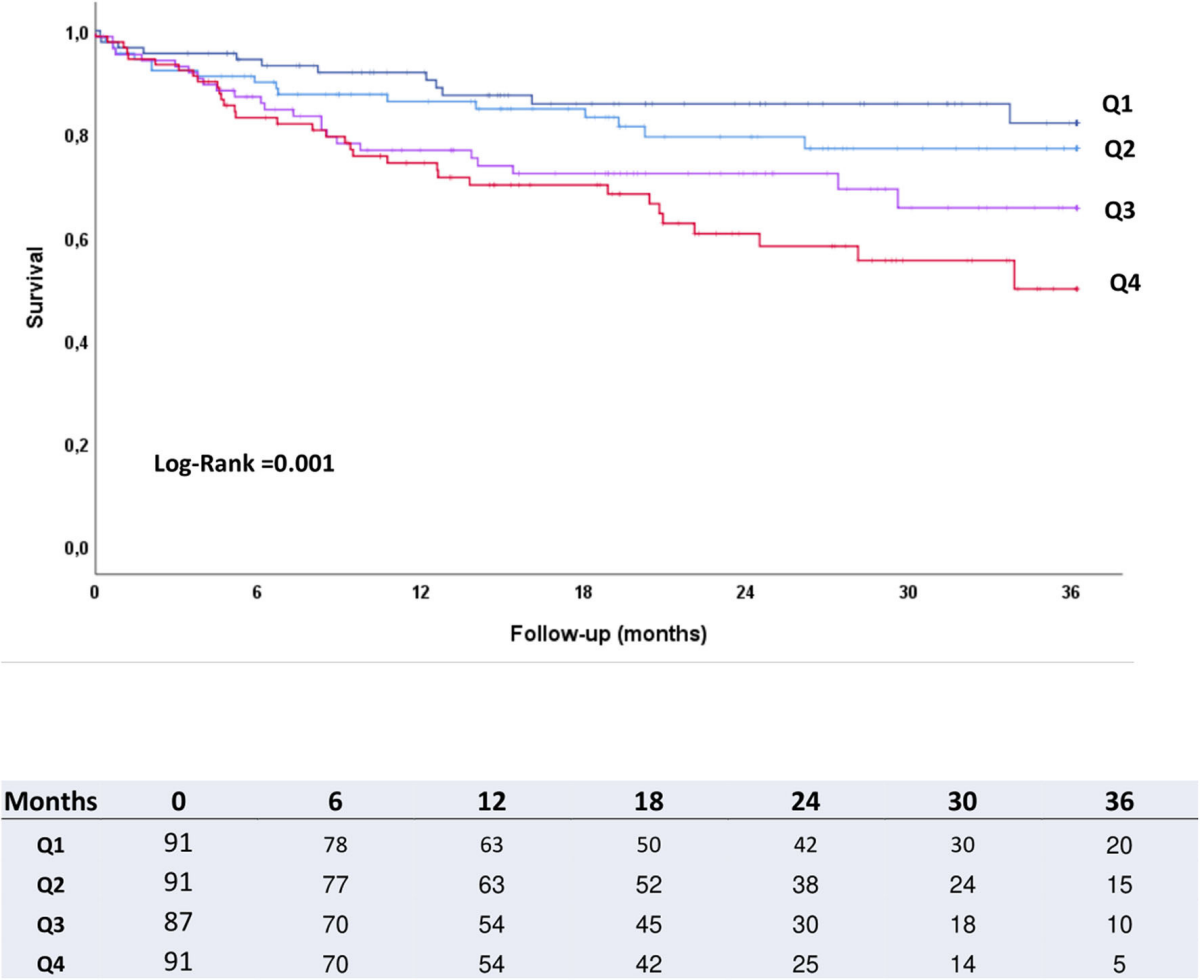
Kaplan-Maier survival curves according to baseline EPO quartiles.

Kaplan-Meier curves revealed that the higher the EPO quartile, the higher the mortality over the study period. As illustrated in Figure 1, curves started to diverge at about 100 days after TAVR, with differences consistently increasing along the subsequent entire follow-up period. In a sensitivity analysis, results showed the same pattern both in male and female patients when analyzed separately, but only achieved statistical significance in female (HR 1.11 [1.01–1.02], log-rank, *p* < 0.001). There was no association between high levels of logEPO and complications according to VARC-2 criteria.

**Figure 2:**
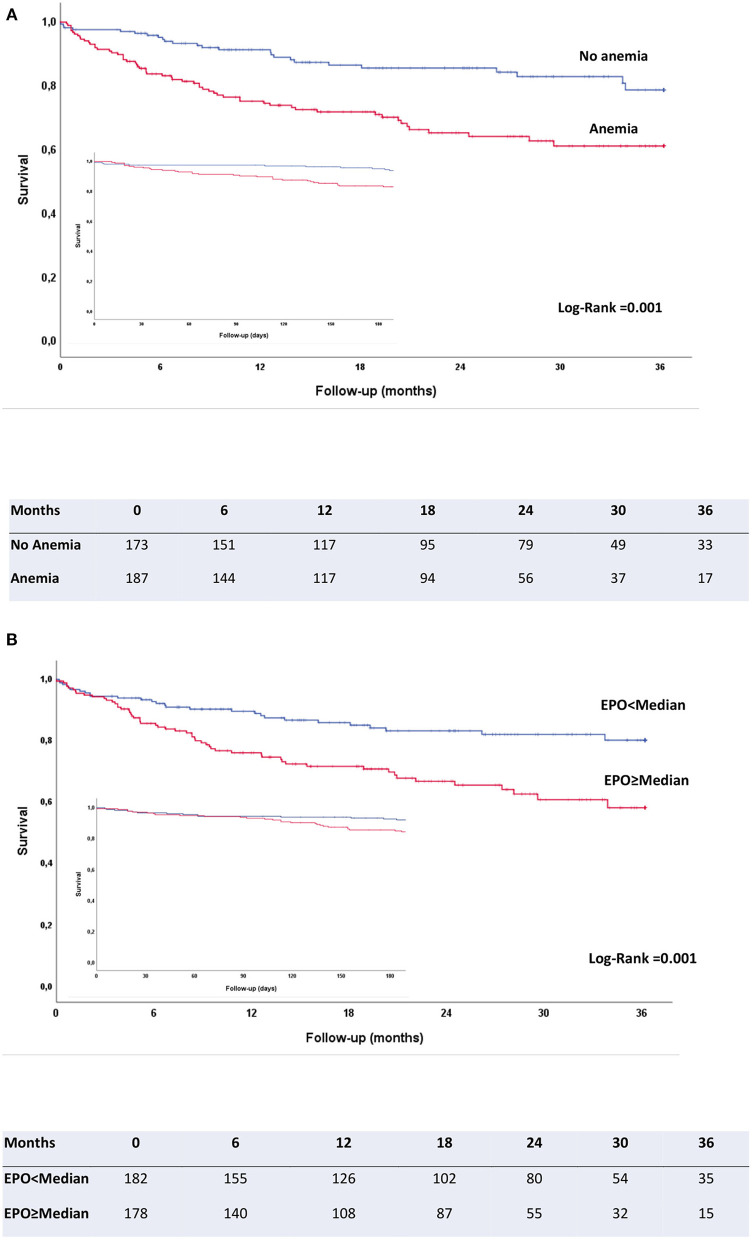
Kaplan-Meier survival curves for patients without and with anemia **(A)** and for patients with EPO values above/below the median EPO level **(B)**. Insert: Kaplan-Meier survival curves for the same groups in the first 180 days after the procedure, to illustrate that anemia/no anemia curves diverge early after the procedure, whereas EPO groups only start to diverge after a few months.

Baseline anemia was also significantly associated with mortality (HR 2.40, 95% CI 1.51–3.80, *p* < 0.001), with Kaplan-Meier curves for anemia vs. non-anemia already diverging shortly after TAVR procedure, as opposed to EPO curves (see Figure 2). On the contrary, baseline iron deficiency had no impact on all-cause mortality after TAVR. Kaplan-Meier curves were very similar in patients with or without statin use (log rank, *p* = 0.919).

All immediate and post-procedural deaths (*n* = 14 up to 30 days) were related to the intervention, with half of them (*n* = 7) directly resulting from procedural complications (including hemorrhagic shock due to vascular access complications or rupture of annulus, rhythm disorders with pulseless electrical activity, and cardiogenic shock) and others being mainly due to stroke, acute renal failure, or in-hospital acquired infection. No EPO-related trends were observed. There were 72 mid-term deaths, with only 50% being due to cardiovascular events (mainly decompensated or terminal heart failure) and others most commonly resulting from cancer (lung, prostate) or infections (pneumonia). Only two mid-term deaths were due to renal failure. No differences in cardiovascular or other causes of death were observed across EPO quartiles.

### Univariate and Multivariate Regression for Mid-term Mortality

In univariate analysis, logEPO levels significantly predicted mortality (HR 4.05, 95% CI 2.29–7.16, *p* < 0.001). All variables showing a *p* < 0.05 for the association with mortality in univariate analysis (see Table 3) as well as an additional clinically relevant variable (i.e., eGFR) were included in a multivariate analysis (see Table 3). A multivariate Cox proportional hazards model showed that higher logEPO levels remained significantly associated with mortality (HR 2.25, 95% CI 1.09–4.66, *p* = 0.029). Specifically, the association remained statistically significant after adjusting for hemoglobin and renal function. In the adjusted Cox model, STS score was the only independent predictor for mortality apart from logEPO level (see Table 3). In a confirmatory separate model including traditional risk factors (age, sex, COPD, hypertension, diabetes), logEPO remained significantly associated with mortality (*p* < 0.0001).

**Table 3:** Cox proportional hazards model for cumulative mortality in overall population.

	**Univariate**		**Multivariate**	
	**HR (95% CI)**	***P*-value**	**HR (95% CI)**	***P*-value**
Log EPO	4.05 (2.29–7.16)	** <0.001**	2.25 (1.09–4.66)	**0.029**
Age (years)	0.99 (0.95–1.04)	0.809	Not selected	–
Sex	1.09 (0.71–1.68)	0.681	Not selected	–
Atrial fibrillation	1.75 (1.14–2.69)	**0.010**	1.38 (0.87–2.18)	0.172
Hypertension	0.72 (0.40–1.31)	0.284	Not selected	–
Diabetes	1.10 (0.71–1.72)	0.661	Not selected	–
COPD	1.31 (0.78–2.20)	0.305	Not selected	
LVEF (%)	0.97 (0.96–0.99)	** <0.001**	0.99 (0.97–1.01)	0.312
STS score	1.12 (1.08–1.16)	** <0.001**	1.09 (1.04–1.15)	** <0.001**
Hemoglobin (g/dl)	0.78 (0.70–0.87)	** <0.001**	0.92 (0.80–1.06)	0.260
eGFR pre (ml/min/1.73 m^2^)	0.99 (0.98–1.00)	0.113	1.01 (0.99–1.02)	0.349
Log NTproBNP (pg/ml)	2.32 (1.55–3.48)	** <0.001**	1.30 (0.74–2.28)	0.369
High-sensitive Troponin T (pg/ml)	1.01 (1.00–1.02)	**0.024**	1.00 (0.99–1.00)	0.762
C-reactive Protein (mg/dl)	1.06 (1.01–1.10)	**0.008**	1.02 (0.96–1.08)	0.443

*COPD, chronic obstructive pulmonary disease; EPO, Erythropoietin; eGFR, estimated glomerular filtration rate; LVEF, left ventricular ejection fraction. P-values in bold are statistically significant*.

Median (IQR) level of EPO in the subset of anemic patients (*n* = 187) was 20 (12.2–34.9) mIU/mL. The previously described O/P ratio was calculated in this subgroup. The majority of patients showed a lower than expected level of EPO production (72.7%) based on their O/P ratio; 15.5% had levels as expected; and 11.8% showed higher than expected levels of EPO. However, higher than expected levels of EPO did not correlate with mortality.

### EPO Impact on Mortality in Selected Subgroups

**Figure 3:**
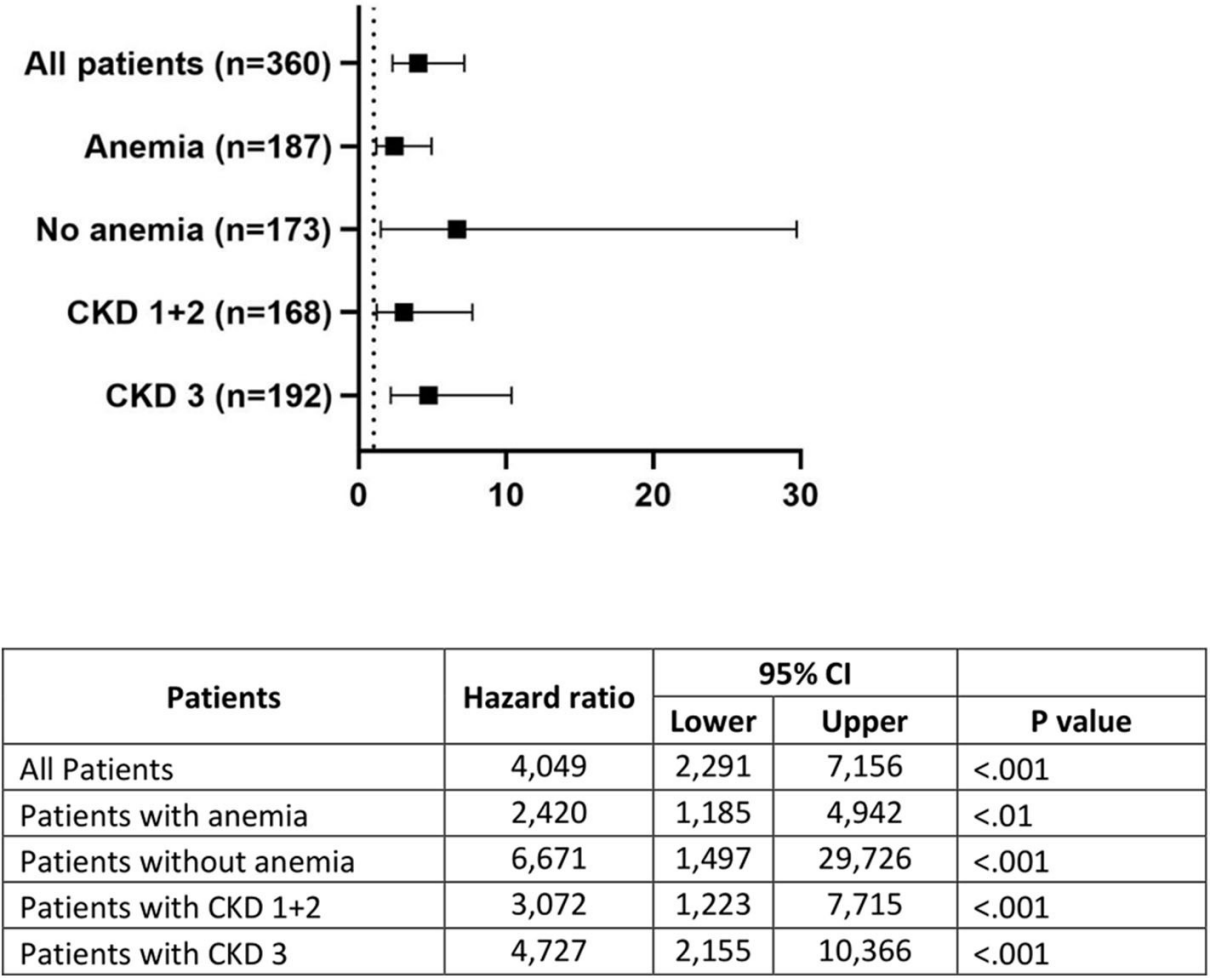
Hazard ratios and 95% confidence intervals of LogEPO in subgroups. CKD: chronic kidney disease stage.

An exploratory subanalysis on the impact of EPO levels on mortality was performed in patients without and with anemia, as well as in patients with normal (CKD 1+2) and moderately impaired renal function (CKD 3) (see Figure 3.) Results were fully consistent in all subgroups, with logEPO significantly predicting mortality in all of them. Interestingly, the highest HR value (6.67 [1.50–29.73]) was observed in non-anemic patients.

## Discussion

To our knowledge, this is the first study to demonstrate that high baseline EPO levels are a significant predictor for mid-term mortality in patients with severe aortic stenosis undergoing TAVR. Importantly, a clinically relevant multivariate HR value (HR 2.25, 95% CI 1.09–4.66, *p* = 0.029, Table 3) was found to be statistically significant after adjusting for a number of important risk factors (particularly anemia and renal function). In exploratory analyses, findings were also consistent in several relevant subgroups, such as patients with/without anemia, and mild-to-moderate renal dysfunction (Figure 3). Several clinical, echocardiographic, and laboratory parameters have been shown to have some prognostic value in severe aortic stenosis (19). However, there is still a need for powerful prognostic biomarkers that can help us at identifying patients with poor prognosis even in the TAVR era and at paving the way to explore novel pathophysiological mechanisms in severe aortic stenosis. If our findings are confirmed in future studies, EPO could be considered as a potentially contributing biomarker in patients not having a severe renal dysfunction.

As previously observed (20, 21), anemia was also a strong predictor for both early and late mortality in our study. However, differently from anemia, the independent prognostic value of EPO appears to emerge only after post-TAVR recovery is completed. This can be clearly observed in Kaplan-Meier curves for the first months after TAVR (Figure 2). One potential explanation for this could be that anemia may have both a mid-/long-term and a short-term deleterious effect through an impact on severe peri-/post-procedural bleeding/vascular complications, whereas high EPO levels may not be associated to immediate complications but only to later clinical course. In fact, several previous investigations have shown baseline anemia to be associated with a higher number of post-TAVR complications (vascular, bleeding), causing a higher mortality in the short-term (22). In spite of this, HR values for cumulative mortality were higher for EPO than for anemia (HR 4.05, 95% CI 2.29–7.16, *p* < 0.001 for logEPO vs. HR 2.40, 95% CI 1.51–3.80, *p* < 0.001 for hemoglobin). As opposed to previous studies in patients with severe aortic stenosis undergoing TAVR (23), baseline iron deficiency did not have an impact on mortality in our cohort. Causes of both post-procedural and mid-term deaths showed no differences across EPO quartiles, suggesting EPO impact was not directly related to TAVR complications. This is consistent with our finding of Kaplan-Meier curves for EPO quartiles only starting to diverge after the immediate post-procedural period. Renal failure was very uncommon as a cause of mid-term death, which supports our findings of EPO impact being independent from renal function.

Renal function (as measured by CKD stage) is also a well-known prognostic factor. As in other studies on EPO (24), in our population, patients with eGFR < 30 mL/min/1.73 m^2^ were excluded because their erythropoiesis is severely impaired and is likely to result in altered EPO levels. Prognostic value of EPO in our study was independent from mild-to-moderate renal dysfunction. Our results are therefore not applicable to patients with severe kidney failure. It should be highlighted, however, that patients excluded for severe kidney failure account for only 9.8% (39/399) of patients in our severe aortic stenosis population.

In this context, the finding of a most pronounced EPO effect in patients with no baseline anemia (HR 6.67, 95% CI 1.50–29.73, *p* < 0.001, Figure 3) is particularly interesting. It would appear that high endogenous EPO confers a higher mortality risk that is not directly related to anemia or mild-to-moderate renal dysfunction and cannot be explained by immediate general operative risk (as assessed by STS score), relevant specific heart disorders such as atrial fibrillation, age, key cardiac laboratory biomarkers such as NTproBNP, and hs-CRP, an inflammatory marker.

In patients with anemia, observed EPO levels were lower than expected in 73% of patients, whereas 12% of patients had higher than expected levels of EPO. This trend has been previously observed for EPO in anemia in other populations, such as heart failure patients (7). However, contrary to such studies, no association of higher-than-expected EPO levels with mortality was observed in our study in aortic stenosis. This could be due to the limited number of patients, but could also suggest distinct mechanisms underlying the impact of EPO on clinical outcomes in patients with or without anemia (24).

The fact that elevated baseline EPO levels are associated with a worse mid-term prognosis in severe aortic stenosis is in line with findings from previous studies in patients with chronic (6) or decompensated heart failure (8); and this could suggest an overall association with heart conditions (i.e., ischemic or dilated cardiomyopathy), not limited to aortic stenosis. Furthermore, high EPO levels were also associated with mortality in an overall elderly population in the Leiden-85-plus study (24). Since severe aortic stenosis requiring TAVR mainly occurs in old patients (median age in our population was 83 years; similar to other series), this is also a factor to be taken into account.

At present, we can only speculate on several potential pathophysiological mechanisms, beyond anemia and renal function, that can be proposed to explain our findings. Firstly, taking into account that low oxygen availability triggers EPO production in the kidney, chronic hypoxia itself, due to a subclinical disorder even with no anemia, rather than renal dysfunction, could elevate EPO levels as a physiologic response (24–26).

Secondly, in our study, although being prognostically independent, EPO levels showed some association with NTproBNP levels and NYHA functional class. In fact, the higher the EPO quartile, the higher the levels of NTproBNP and the proportion of NYHA class IV patients (see, Table 1). This finding was previously observed in patients with chronic heart failure by Van der Meer et al., who found EPO levels to be correlated with severity of chronic heart failure (6). Elevated EPO levels could in some way reflect a more advanced state of decompensation due to valvular obstruction, which could result, symptomatically, in an advanced NYHA stage and, more objectively, in higher levels of NTproBNP. Maybe EPO could represent a biomarker encompassing several pathophysiological aspects that could help us detect patients with an advanced disease status when considering a high-risk TAVR procedure.

Thirdly, in an elderly population, bone marrow senescence and exhaustion could lead to elevated EPO levels in an attempt to overcome a potential bone marrow resistance (18, 24) in a proinflammatory state (7). However, such relative EPO deficiency would imply rhEPO use could be beneficial (15), which has not been the case in clinical studies in heart failure (3, 4). EPO is also related to inflammatory processes (27). However, EPO prognostic value in our cohort was independent from hs-CRP, an inflammatory biomarker. More detailed studies on the relationship between EPO and inflammation are needed in this population.

Fourthly, EPO is synthetized by the kidney not only as a response to anemia or hypoxia, but also when stimulated by reduced renal perfusion caused by low cardiac output (15). Thus, it could be worth to specifically investigate the impact of endogenous baseline EPO levels in patients with a low-flow low-gradient aortic stenosis, since this population can be characterized by a reduced cardiac output.

Lastly, although it appears difficult to find plausible mechanisms to explain it, EPO impact could also be specifically related to the effects of TAVR therapy itself. This seems, however, very unlikely, because EPO levels showed no association with TAVR complications, and EPO prognostic impact only emerged a few months after TAVR once post-procedural recovery was completed.

### Study Limitations

This is an exploratory study and findings should be taken as hypothesis-generating. In interpreting our findings, we acknowledge as a limitation that patients with severe aortic stenosis not undergoing TAVR were not included in our study. However, in our center, few patients are excluded from the procedure, usually because of their very poor short-term prognosis or comorbidities resulting in an extremely high procedural risk. Such few patients have a low short-term survival and would not have influenced in the mid-/long-term results. And including them in our analysis would have interfered also with the interpretation of the short-term results potentially related to the TAVR procedure itself. Patients undergoing surgical aortic valve replacement were also excluded. The effects of the significant acute surgical insult and the required postsurgical therapy make them a distinct population with their prognosis being dependent on other factors.

This is a single center study; thus, extrapolation to other populations needs to be performed cautiously. Our results are not applicable to patients with severe renal dysfunction (accounting for 9.8% of patients in our cohort), as they were excluded from our analyses. Although we report a mid-term follow-up of up to 3 years, cause of mortality was not always available and could not be specifically evaluated.

### Future Insights

Whilst endogenous EPO levels have been shown to predict survival in this cohort, it remains to be studied whether EPO is also a useful marker for monitoring, follow-up and clinical management of patients with severe aortic stenosis after TAVR, as has been observed in a few studies in patients with heart failure (8). Additional post-baseline EPO measurements could provide us with better insights into the prognostic usefulness of EPO.

## Conclusions

Differently from anemia, which was a strong predictor for both early and late mortality in patients with severe aortic stenosis undergoing TAVR, high baseline EPO levels were an independent biomarker for mid-term mortality, with its prognostic value only emerging after post-TAVR recovery was completed. EPO prognostic value was independent from anemia and mild-to-moderate renal dysfunction. EPO levels add to the existing prognostic biomarkers in patients with severe aortic stenosis in the TAVR era.

## Data Availability Statement

The raw data supporting the conclusions of this article will be made available by the authors, without undue reservation.

## Ethics Statement

The studies involving human participants were reviewed and approved by Goethe University Frankfurt am Main. The patients/participants provided their written informed consent to participate in this study.

## Author Contributions

SM-P, SF, and MV-N conceived and designed the study. SM-P and JY acquired the data. SM-P and AB analyzed the data. PS, RD, and M-IM assisted in the analysis and interpretation of the results. SM-P and MV-N drafted the manuscript. SM-P, PS, RD, M-IM, JY, SF, MV-N and AZ critically revised the manuscript for intellectually significant content. All authors read and approved the submitted version.

## Conflict of Interest

SF was proctor and reports consultancy activities for Abbott Vascular and Edwards Lifesciences. MV-N was proctor for Abbott Vascular, Medtronic, Edwards Lifesciences and Boston Scientific. The remaining authors declare that the research was conducted in the absence of any commercial or financial relationships that could be construed as a potential conflict of interest.
